# Investigation of Phosphate Removal Capability of Blast Furnace Slag in Wastewater Treatment

**DOI:** 10.1038/s41598-019-43896-y

**Published:** 2019-05-16

**Authors:** Sara Yasipourtehrani, Vladimir Strezov, Tim Evans

**Affiliations:** Department of Environmental Sciences, Faculty of Science and Engineering, Macquarie University, Macquarie, NSW 2109 Australia

**Keywords:** Pollution remediation, Environmental impact

## Abstract

Blast Furnace Slag (BFS) is a by-product of iron making with a potential to be used in different applications. In this research, BFS is used to investigate the phosphate removal ability in wastewater. BFS has the required concentrations of surface calcium to potentially precipitate phosphate from wastewater. Removal of phosphate from wastewater depends on variety of conditions, such as the size of BFS particles, adsorbent dose, contact time and pH. The conditions responsible for phosphate removal from wastewater with BFS were analysed and the phosphate removal capacity optimised according to the BFS chemical content. The results in this work demonstrated that the basicity (CaO/SiO_2_) of BFS has a reverse effect on phosphate removal capacity. High basicity reduces the capability of BFS for removal of phosphate. BFS composition before and after phosphate removal was determined with Energy Dispersive Spectroscopy (EDS), Fourier Transfer Infrared Spectroscopy (FTIR) and UV-Vis spectrophotometry. The results revealed that the slag samples added varying concentrations of trace metals Al, Cd, Co and Hg into the treated water, which will need to be further conditioned by dilution with unpolluted water or other treatments before disposal or re-use.

## Introduction

Blast Furnace Slag (BFS) is the typical waste product produced in significant amounts of 270–320 Mt per year worldwide by the ironmaking industry^1^. The main components of BFS are lime, silica, alumina and magnesia. Although considered as a waste product, the slag has specific properties and chemical content that makes it valuable in several applications. According to the European Commission, management of by-products of the industry is important for the circular economy, therefore emphasis is placed to investigate alternative methods for waste management of industry by-products^2^. The reuse of BFS as a part of other industries reduces environmental contaminants, energy consumption^3^ and production costs^2,4^. One of these potential applications is the use of BFS for removal of phosphates in wastewater.

Phosphate is produced and released from different processes, such as fertilizer production, mining and minerals processing, textile and leather processing, pigment formulation and wastewater treatment^5^. The concern with phosphorous as a water pollutant became serious after 1970s due to its significant eutrophication impacts from uncontrolled discharges into the water body, instigating worldwide changes to the water discharge legislations^6^. The concentration of phosphate produced by industry and discharged through their wastewater streams is now controlled^7^, however, it is important to consider the cumulative effect of contaminants released in the same natural ecosystem^8^. The Australian and New Zealand guidelines for fresh and marine water quality state that to ensure the protection of aquaculture species, phosphate concentration from primary industries should be <0.1 mg/L in freshwater and <0.05 mg/L in salt water^8^.

Phosphorus in wastewater is a major threat as it provides nutrient pollution for growing harmful organisms (algal growth) in the natural ecosystems and causes their deterioration and euthrification by reducing the dissolved oxygen^6,9–11^. Depletion of oxygen is harmful to aquatic life and decreases biodiversity. It also affects the recreational value of each ecosystem^5^.

There are three main methods for removing phosphate from wastewater, consisting of physical, biological and chemical treatments^2,12^. The physical mechanisms consist of microfiltration, reverse osmosis, electrodialysis and magnetic separation^13^. However, the physical treatment is not sufficient for complete phosphate removal, as it is expensive and removes only 10% of the total phosphate. The biological mechanism can be classified into assimilation, enhanced biological P removal, constructed wetlands and wastewater stabilization ponds. The constructed wetland systems were established as a promising way to remove phosphate from wastewater, however, the important part of these wetlands are the materials used as a wetland substrate^6^. The removal mechanism of phosphate by the wetland systems depends on the amount of iron, calcium or aluminium in the substrate. In addition, other properties, such as hydraulic conductivity, porosity, granulometry, and the surface area have impact on the ability of phosphate removal^14^. The biological methods remove up to 97% of the total phosphate but they are difficult to operate and can cause fluctuation in temperature and chemical content of the wastewater^9,12,15^. The chemical processes are divided into precipitation, crystallization, anion exchange and adsorption. The technologies based on chemical dosing or activated sludge can also be integrated after the biological wetland or pond systems to improve the phosphate removal efficiencies. The chemical method is widely used as it is reliable and economical in comparison with the other two methods^9,12^. The chemical removal of phosphates through precipitation consists of conversion of the soluble phosphate into insoluble form using chemicals and then removal from the water through precipitation. Alum salt is one of the common additives that can convert the soluble phosphate into insoluble aluminium phosphate^16^. Inorganic sorbents are promising chemical methods to increase removal activity by using industrial wastes, such as BFS and fly ash^17^. Inorganic sorbents can additionally remove heavy metals and organic pollutants^18^. For removing phosphate from wastewater, high amounts of calcium, aluminum and iron are required^17^. The advantage of wastewater treatment through adsorption is the low cost, high efficiency and is easy to operate^13^.

Metallurgical slags, including BFS, have been used to remove phosphates from wastewater through a physical and chemical adsorption mechanism^19–23^, consisting of precipitation and ion exchange with small physical interaction between the surface of the sorbent and the metallic salt of phosphorous^15,23^. Agyei *et al*.^24^ demonstrated PO_4_^3−^ removal through chemisorption using fly ash and slag. The chemical mechanism of phosphate removal from wastewater with BFS occurs by hydration reaction of slag in a water phase by increasing the pH and producing the calcium oxide, magnesium oxide, calcium silicate and calcium aluminate^21^. The physical mechanism occurs by negative charge of the slag surface. Alkaline conditions promote negative charge on the slag surface, which repulses the negatively charged species in the solution^25^. Under high pH conditions, the phosphate adsorption is low because of the repulsion between negative charged surface area of slag and negatively charged PO_4_^3–25^. When BFS is used for phosphate removal, the amount of Ca in BFS decreases as precipitation of Ca-P occurs^23^.

The chemical content of the slag surface affects the phosphate removal ability. BFS has two phases, namely amorphous and crystalline, which are formed during the cooling process^20^. According to Johansson^19^, the crystalline slag shows a tendency for sorption of phosphate, while the amorphous slag does not show any tendency for phosphate removal. The common oxides contained in BFS can act in water as acidic (SiO_2_), alkaline (CaO) or amphoteric (MgO, Al_2_O_3_)^20,26^. The mechanisms of phosphorous removal by BFS have been studied, showing that the calcium-based fraction present in BFS was the active component responsible for dissolving phosphorous^15^. Kuwahara & Yamashita *et al*.^27^ prepared calcium silicate hydrate from BFS and applied batch experiments to monitor the phosphate removal ability of BFS. This study demonstrated that the synthesized BFS is able to remove phosphate 73 times greater than the normal BFS. However, batch experiments showed that BFS has high efficiency for phosphate removal^28^. Temperature, agitation rate, adsorbent dose and pH affect the adsorption of phosphate by BFS^9^.

Large amount of calcium and alkaline conditions (pH > 9) are required to remove phosphorus^28^. When the pH is above 10 the main mechanism is chemical precipitation, while in the pH is below eight physical adsorption is the main retention mechanism^29^. The size of slag particles has an important effect on phosphate adsorption, as the fine slag particles (<0.1 mm) adsorb phosphate better^18^. According to Kostura *et al*.^29^, when the particle size of the slag decreases from 1.5 µm to 0.2 µm the specific surface area of slag increase 27 times and the adsorption capacity increase 127%.

Different studies showed the ability of BFS to remove phosphate from wastewater^9,15,18,19,27–29^; however, the impact of the slag basicity on the phosphate removal efficiency is still not well understood and the leaching behaviour of BFS and the spices that introduce to the wastewater after subjecting to the BFS to remove phosphorous. The aim of this work was to study the effect of the chemical content and basicity of BFS on its ability to remove phosphate from wastewater.

## Materials and Methods

### Materials

**Figure 1:**
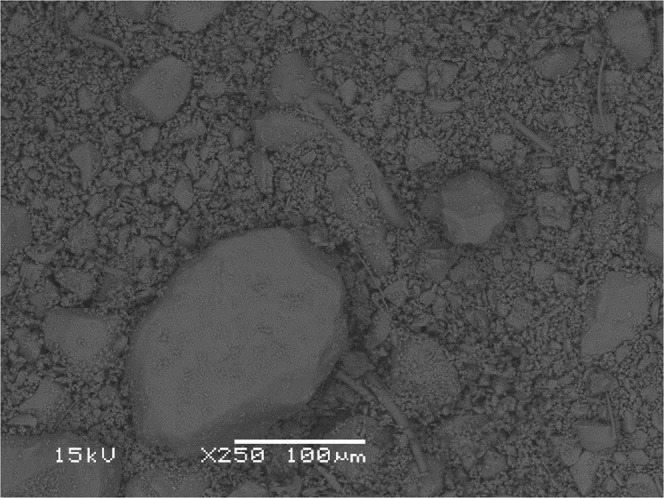
The size of sample R powder after grinding according to the SEM imaging.

Three BFS samples used in this study, identified as A, B and R, were supplied from three different ironmaking blast furnace (BF) plants in China. To minimise variability of the samples, each BFS was ground by using a standard ring mill batch pulveriser to form homogenous sub-sample and reduce the particle size to have the sample with same chemical content, and hence increase its adsorption capability. The size of BFS particles after grinding was determined with SEM imaging and it was determined by averaging 10 areas of each sample with the magnification of 250 times. The result revealed that the particle size distribution was between 10–100 µm (Fig. 1). The average chemical analysis of the samples, determined with X-ray fluorescence spectroscopy, is presented in Table 1, while pH was determined by a pH meter. For calculating the pH of the BFS samples, 1:5 (w/v) of BFS was diluted with deionised water and shaken for 24 hours. Basicity was calculated as the ratio of CaO/SiO_2_ and was found to vary between the samples. In addition, all three samples are coarse amorphous and have glassy phase due to the rapid cooling of the molten slags.

**Table 1:** Chemical composition of the samples.

Slag	TFe	SiO_2_	CaO	MgO	Al_2_O_3_	MnO	TiO_2_	Basicity	pH
Sample A	0.43	35.93	40.41	8.38	13.54	0.26	0.5	1.12	11.04
Sample B	0.35	33.31	40.94	8.46	14.99	0.26	0.57	1.22	11.47
Sample R	0.39	30.78	33.83	13.34	19.01	0.84	0.97	1.09	11.83

Synthetic wastewater was prepared by diluting a standard phosphate solution (1000 mg P/L) from Hach with deionised (DI) water to prepare wastewaters with different concentrations of phosphate (100, 150, 180, 210 and 250 ppm).

## Methods

### Batch experiment

Batch experiments were undertaken to determine the capacity of phosphate sorption of each BFS. Batch experiments have typically been applied in previous studies to determine the ability of phosphate removal by BFS^27,29,30^. In the current study, different conditions were considered to determine the optimum requirement to remove the highest amount of phosphate from wastewater.

#### Adsorbent dose

Adsorbent dose (BFS concentration) was used in the range between 40–80 g/L to determine the optimum concentration of BFS for removing phosphorous. The phosphate concentration was kept in all samples constant at 100 ppm to monitor the effect of adsorbent dose for removing the highest amount of phosphate from wastewater. The samples were shaken for one hour in a shaker with the speed of 10 rpm and then the BFS was separated from the liquid by a 45 µm filter paper. The phosphate concentration after treatment with BFS was measured with Vis spectrophotometry. One duplicate analysis was performed to validate the reproducibility of the result.

#### Contact time

To determine the optimum contact time required to remove the phosphate, different contact times of 0.167, 0.5, 1, 2, 4, 8, and 24 hours were applied, in accordance to the study performed by Lu *et al*.^18^. In this step, the optimum adsorbent dose for phosphate removal was used from the previous step and the adsorbate dose was kept the same to confirm the effect of contact time. Phosphate in the wastewater was 100 ppm and the BFS concentration was 60 g/L. The samples were shaken at different times in a shaker with the speed of 10 rpm and then the BFS was separated from the liquid by a 45 µm filter paper. The phosphate concentration after the BFS contact effect was measured by Vis spectrophotometry. In addition, one duplicate analysis was performed to validate the reproducibility of the result.

#### Adsorbate dose

In this step, the phosphate concentration in the wastewater was changed from 100 ppm to 250 ppm. The optimum adsorbent dose determined in the first step was used to determine the optimum concentration of phosphate that could be removed by the BFS samples. The samples with different concentration of phosphate were shaken in a shaker with the speed of 10 rpm and then the BFS was separated from the liquid by the 45 µm filter paper. The phosphate concentration after the BFS contact effect was measured in the Vis spectrophotometry. Two duplicate samples were analysed under comparable conditions to estimate the reproducibility of the results.

### Fourier transform infrared (FTIR) spectroscopy

FTIR was used to investigate the changes of sample spectra when the sample was mixed with different concentrations from 100, 150, 180, 210 and 250 ppm of phosphate in synthetic wastewater. The samples were subjected to FTIR analysis using Nicolet 6700 FTIR spectrometer through total 32 scans with a resolution of 4 cm^−1^.

### Energy dispersive spectroscopy (EDS)

Energy Dispersive Spectroscopy (EDS) analysis was used to measure each BFS composition before and after mixing with different concentrations of phosphate containing wastewater. The EDS was performed on a JEOL6480LA SEM with the pressure used inside the microscope chamber being 30 Pa to avoid electrical charges on the dielectric slag samples. The accelerating voltage was 15 kV and the energy dispersion range was between 0–20 keV at the working distance of 10 ± 2 mm. In this experiment, the result is the average EDS analysis measured within a three square map readings of the EDS of the BFS samples from each BF plants.

### Trace element analysis

The samples were sent to the National Measurement Institute for measurement of trace elements using inductivity coupled plasma mass spectrometry (ICP-MS). The samples were first digested with HNO_3_-HCl at 95–100 °C for two hours. One blank sample was used for each concentration for calibration purposes.

## Result and Discussions

### Batch experiments

Batch experiments were used to determine the optimum conditions for removal of phosphate from the wastewater. According to the pH of the BFS samples presented in Table 1, all samples had pH above 9 and produced the alkaline conditions theoretically needed for removing the phosphate^28^. The batch experiments were designed to determine the adsorbent dose, adsorbate dose and contact time.

#### Adsorbent dose

The most effective adsorbent dose to remove phosphate from the synthetic wastewater was determined by changing the amount of BFS in contact with the wastewater while maintaining the remaining conditions constant.Three different concentrations of adsorbent (BFS) at 40, 60 and 80 g/L were used in this study. The synthetic wastewater had phosphate concentration of 100 ppm while the contact time was 1 hour shaken with the speed of 10 rpm. The concentration of phosphate in wastewater was measured after subjecting and filtering the BFS by Vis spectrophotometry. Table 2 presents the concentration of wastewater with 100 ppm phosphate after contact with BFS at different concentrations (40, 60 and 80 g BFS/L). The optimum result was achieved with BFS concentrations of 60 g BFS/L, resulting in the phosphate concentration in the wastewater being reduced by approximately 15–20%. BFS at 40 and 80 g/L reduced the phosphate concentration between 8–17% and 13–19% respectively. The result of the duplicate sample showed 0.21% reproducibility of the results.

**Table 2:** Adsorbent dose change effect on phosphate removal.

	40 g BFS/L	60 g BFS/L	80 g BFS/L
Sample A	82.7 mg/L PO_4_^3−^	80.3 mg/L PO_4_^3−^	80.8 mg/L PO_4_^3−^
Sample B	91.3 mg/L PO_4_^3−^	84.2 mg/L PO_4_^3−^	86.9 mg/L PO_4_^3−^
Sample R	83.1 mg/L PO_4_^3−^	82.2 mg/L PO_4_^3−^	83.6 mg/L PO_4_^3−^

#### Contact time

The effect of contact time on phosphate removal was determined by changing the contact time from 0.167, 0.5, 1, 2, 4, 8 and 24 hours, while maintaining 60 g BFS/L as the optimum concentration of BFS to remove the phosphate from wastewater. In Table 3, the phosphate removal efficiency is presented. In the first hour of mixing and shaking of BFS and wastewater the highest amount of phosphate removal occurred, while after 1 hour, the reduction of phosphate did not change significantly in all three samples which is in accordance to the observations by Lu *et al*.^18^. As shown in Table 3, sample A with 29.3% of phosphate removal had the highest impact and sample B with 15.8% had the lowest impact on removal of phosphate in the first hour of reaction. In addition, sample R was able to remove 17.8% of phosphate from the wastewater during the same period of time. According to the data, 1 hour contact time was considered suitable for further tests as the phosphate concentration was appropriately reduced at this contact time. The duplicate sample confirmed the acceptable reproducibility of 0.07% in the result under constant conditions of the analysis.

**Table 3:** Contact time effect on the percentage of phosphate removal.

	Sample A (%)	Sample B (%)	Sample R (%)
10 minutes	10.9	9.47	10.7
30 minutes	17.7	14.98	16.1
1 hour	19.7	15.8	17.8
2 hours	18.63	14.1	15.3
4 hours	19.6	15.1	16.08
8 hours	19.02	15.3	16.9
24 hours	19.22	15.57	16.75

#### Adsorbate dose

**Figure 2:**
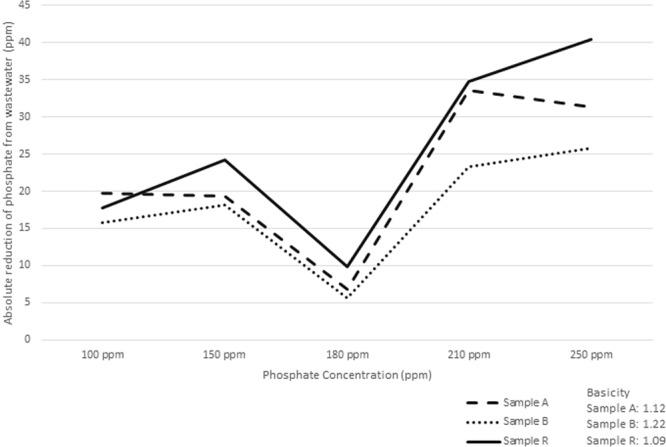
Phosphate reduction in contact with the same amount of BFS for three samples.

The adsorbate dose or the effect of wastewater phosphate concentration on the BFS phosphate removal efficiency was determined using 60 g BFS/L and 1 hour contact time for all samples, while the concentration of phosphate in the synthetic wastewater was changed from 100, 150, 180, 210 and 250 ppm. The results of phosphate concentration after contacting with BFS are presented in Table 4 and Fig. 2, which show the phosphate reduction in the wastewater with different concentrations of phosphate. The greatest phosphate reduction is allocated to the wastewater with 210 ppm for sample A at 33.5 mg PO_4_, and the concentration of 250 ppm for sample B and R at 25.75 mg PO_4_ and 40.44 mg PO_4_ reduction, respectively. It has been demonstrated in the past that phosphate removal efficiency depends on the calcium, aluminium and titanium content of the sorbent^31^. For the wastewater with low concentration of phosphate, the major mechanism of sorption is through chemisorption, while for wastewater with high concentration of phosphate the main mechanism is surface precipitation^31^.

**Table 4:** Effect of BFS on removal of phosphate from synthetic wastewater at different phosphate concentrations.

Phosphate Concentration	Sample A (mg/L PO_4_^3−^)	Sample B (mg/L PO_4_^3−^)	Sample R (mg/L PO_4_^3−^)
**Phosphate concentration after contact with BFS**			
100 ppm	80.3	84.2	82.2
150 ppm	130.6	131.9	125.8
180 ppm	173.2	174.4	170.2
210 ppm	176.5	186.75	175.25
250 ppm	218.74	224.25	209.56

The effect of basicity of each sample on phosphate removal ability of BFS was investigated, with BFS sample R having the lowest basicity, while sample B had the highest basicity (Table 1). Figure 2 demonstrates the phosphate removal in each sample with different wastewater phosphate concentrations. Sample R with the lowest basicity, had the highest ability to remove wastewater phosphate, while sample B with the highest basicity had the lowest ability to remove phosphate from the wastewater. Moreover, the two laboratory duplicate analysis shows the measurement reproducibility of laboratory analysis within 0.23% and 0.09%.

### Fourier transform infrared (FTIR) spectroscopy

**Figure 3:**
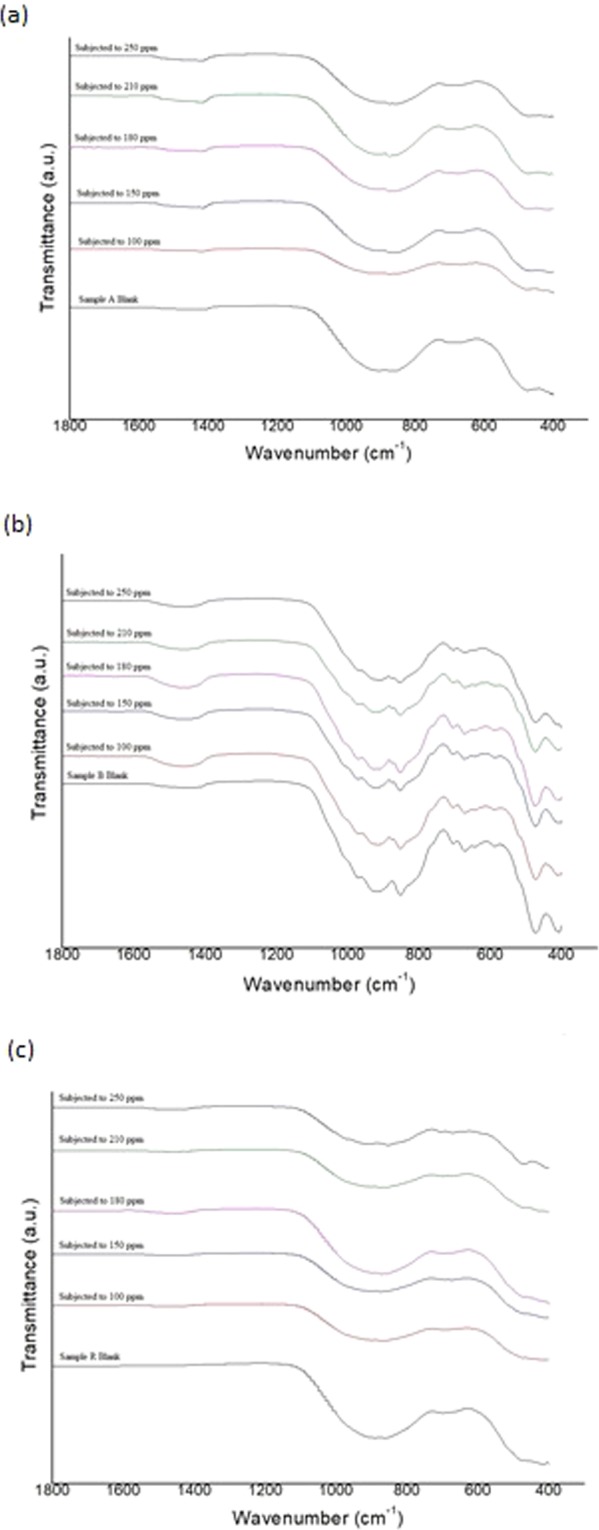
FTIR spectroscopy of blast furnace samples with different concentration of phosphate in synthetic wastewater; (**a**) sample A; (**b**) sample B; (**c**) sample R.

Figure 3 shows the FTIR spectroscopy of the three samples comparing the unreacted (0 ppm) BFS with the slags subjected to wastewater with phosphate concentrations between 100 and 250 ppm. The wavenumber at 1611 cm^−1^ is associated with water bands and OH groups in the sample^32^. The wavenumber range between 1560 and 1360 cm^−1^ is associated with the carbonate CO_3_^2−^ phase^33^ and has shown increase in transmittance for the slag samples after wastewater treatment. The wavenumber at 963 cm^−1^ is related to the antisymmetric stretching vibration of (Al)-O, and the wavenumber at 876 cm^−1^ is related to the symmetric stretch of the AlO_4_^−1^ group, while the 916 cm^−1^ is related [SiO_4_] tetrahedral^32,34^. These wavenumbers are reduced for all three samples after application of BFS for removing the phosphate from wastewater, presenting the effect of Al on phosphate removal ability, where higher amount of Al increases the phosphate removal ability of BFS. Sample R, with the highest amount of Al in comparison to samples A and B, achieved the highest reduction in phosphate removal, according to the batch experiments.

Furthermore, the 711 cm^−1^ wavenumber is related to the symmetric stretching vibration of Si-O-Si (Al) bridge, and 686 cm^−1^ wavenumber is related to Si-O-Al establishment from [SiO_4_] tetrahedral, which is in correlation to the sample basicity (CaO/SiO_2_) ratio^32,34^. The Si-O-Si (Al) vibration in sample R, which has the lowest basicity (1.09) and the highest phosphate removal ability, is reduced when compared to sample B with the highest basicity (1.22) and lowest phosphate removal ability. The typical phosphate anions observed at 539 and 605 cm^−1^ after phosphate adsorption^35^ have shown only limited appearance in the spectra obtained in the current work.

### Energy dispersive spectroscopy (EDS)

Each BFS was subjected to chemical analysis by EDS to determine its changes after exposure to the synthetic wastewater. Table 5 shows the average EDS analysis measured within a square map reading of the EDS of the BFS samples after treatment of the wastewater with phosphate concentrations ranging between 100 ppm to 250 ppm. The EDS data indicates that the amount of SiO_2_ was reduced when the BFS samples were used to treat the wastewater with phosphate and the amounts of CaO increased in contact with the wastewater. The highest rate of phosphate reduction was related to the amount of Ca which is dissolved by the wastewater. Calcium salt can remove phosphates by precipitation and produce calcium phosphate compounds^31^. The pH, calcium and phosphate concentrations have an effect on the form of precipitation and the produced solutions, such as amorphous calcium phosphates (ACPs), dicalcium phosphate (DCP), dicalcium phosphate dihydrate (DCPD), octocalcium phosphate (OCP), tricalcium phosphate (TCP) and hydroxyapatite (HAP)^36^. The capacity of phosphate removal depends on the type of BFS materials, such as coarse crystalline BFS, fine crystalline BFS, coarse amorphous BFS and fine amorphous BFS^31^. The samples used in this experiment are all coarse amorphous BFS that were ground to fine amorphous BFS before the experiments to ensure homogenous samples in their chemical content. Another reason for the removal capacity is in the surface structure that determines the ability of calcium reactivity^31^. The EDS analysis shows increased CaO concentrations at the surface of the particle with increased phosphate concentration in the treated wastewater, mainly due to the precipitation that occurred on the particle surface after removal of the phosphate.

**Table 5:** Changes in the average chemical composition of three samples when subjected to different concentrations of phosphate.

Sample A	MgO	Al_2_O_3_	SiO_2_	P_2_O_5_	CaO	TiO_2_	MnO	FeO
0 ppm	8.38	13.54	35.95	0	40.41	0.5	0.26	0.43
100 ppm	6.78	12.67	29.87	0.18	49.05	0.62	0.25	0.55
150 ppm	7.21	13.17	30.69	0.407	47.13	0.61	0.37	0.39
180 ppm	7.61	13.07	31.68	0.36	46.09	0.7	0.33	0.13
210 ppm	5.09	10.55	24.39	0.39	57.9	0.69	0.49	0.46
250 ppm	11.47	17.73	30.91	0.13	37.34	1.07	0.8	0.51
**Sample B**	**MgO**	**Al**_**2**_ **O**_**3**_	**SiO**_**2**_	**P**_**2**_ **O**_**5**_	**CaO**	**TiO**_**2**_	**MnO**	**FeO**
0 ppm	8.46	14.99	33.31	0	40.94	0.57	0.26	0.35
100 ppm	7.65	14.15	29.13	0.42	47.18	0.68	0.32	0.44
150 ppm	7.45	14.32	29.76	0.7	46.07	0.41	0.47	0.87
180 ppm	7.19	13.53	27.14	0.12	50.13	0.72	0.44	0.69
210 ppm	7.4	15.06	28.23	0.24	47.76	0.69	0.40	0.29
250 ppm	8.56	15.69	30.88	0.35	43.46	0.65	0.22	0.15
**Sample R**	**MgO**	**Al**_**2**_ **O**_**3**_	**SiO**_**2**_	**P**_**2**_ **O**_**5**_	**CaO**	**TiO**_**2**_	**MnO**	**FeO**
0 ppm	13.34	19.01	30.78	0	33.83	0.97	0.84	0.39
100 ppm	12.84	18.58	28.4	0.26	37.37	0.92	1.22	0.35
150 ppm	11.23	17.34	25.92	0.26	42.6	0.9	1.24	0.47
180 ppm	10.2	15.28	22.91	0.23	46.96	1.87	2.14	0.38
210 ppm	11.57	18.22	26.6	0.2	40.34	1.23	1.42	0.38
250 ppm	9.99	15.9	29.74	0.19	42.21	0.88	0.75	0.31

### Trace elements analysis

**Figure 4:**
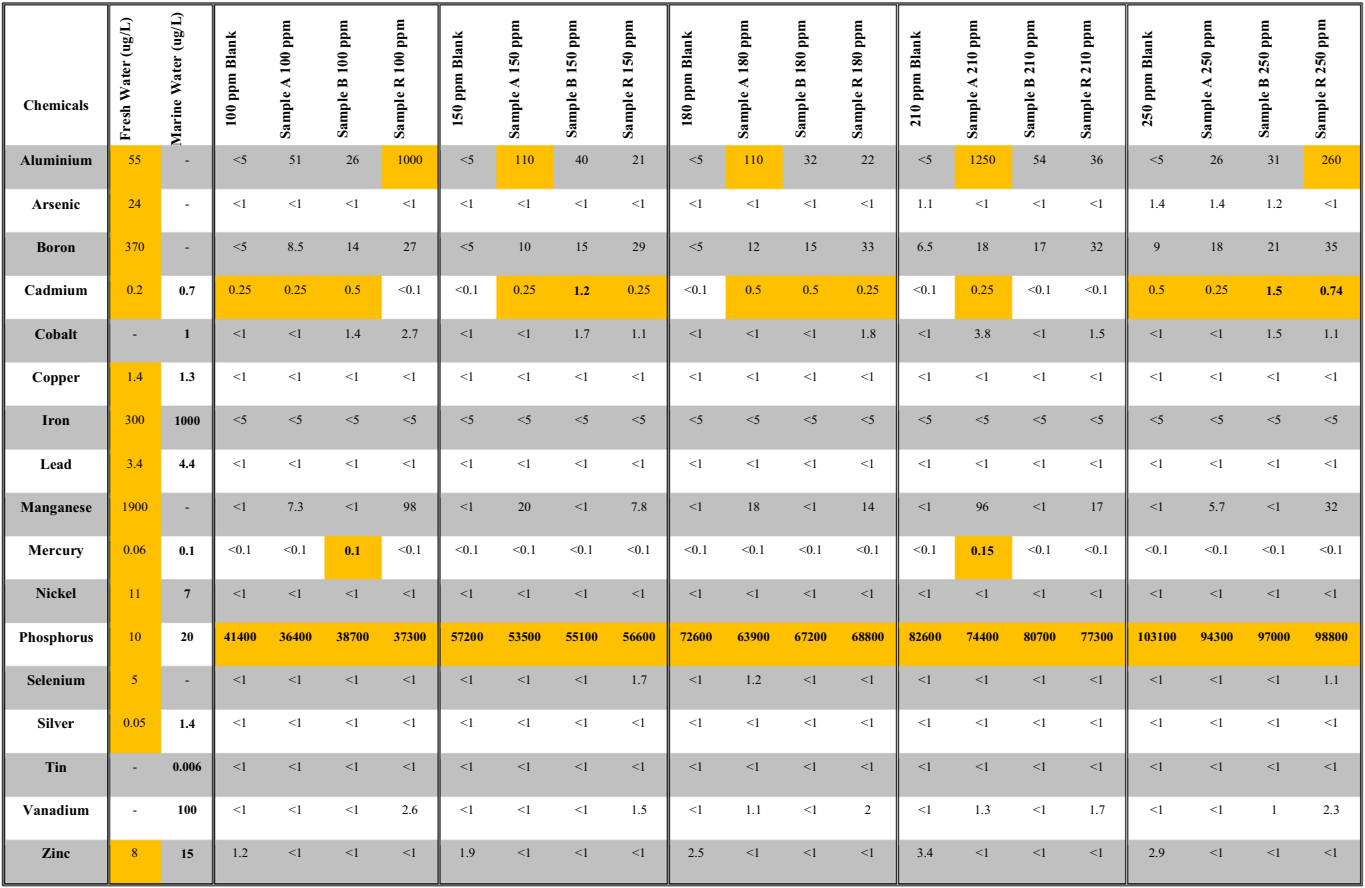
Trace elements analysis.

The results of trace elements analysis are presented in the Fig. 4. This analysis was performed to understand the leaching effect of BFS species and their influence on the quality of water after the mixing process. It is important to determine which elements will be introduced to the water after the phosphate removal activity by BFS. Both the ANZECC and ARMCANZ^8^ were used as a guideline for marine and fresh water acceptable element concentrations. The trace elements, when compared to the guidelines, indicate that aluminium exceeded the fresh water criteria in some of the samples. In addition, cadmium exceeded the guideline for both the fresh water and marine water in some samples. Cobalt is another element that exceeded the guidelines for marine water, and mercury exceeded the guideline values in one sample for both fresh and marine water.

## Conclusions

This study investigated the ability of BFS to remove phosphate from wastewater considering physical conditions, such as adsorbent (BFS) dose, contact time and adsorbate (wastewater phosphate concentration) dose; and chemical conditions such as chemical content of BFS and the basicity. The results showed that the optimum adsorbent dose of BFS is 60 g/L and the optimum contact time is 1 hour. The changes of adsorbate dose were further investigated and the highest percentage of phosphate that can be removed by BFS was determined to be 100 ppm. According to the EDS analysis, all three BFS samples after subjecting to wastewater treatment decreased in the concentration of SiO_2_ and increased in CaO. The CaO increased due to diffusion of the particle to the surface, and SiO_2_ decreased by dissolution into the wastewater. In addition, the FT-IR analysis showed the vibration in 963 cm^−1^ wavenumber related to the Al-O bond, vibration in 711 cm^−1^ wavenumber which is related to the Si-O-Si (Al) bridge and 494 cm^−1^ that is related to the O-Si-O bond vibration which have an effect on phosphate removal ability of BFS. The comparison of the BFS basicity and phosphate removal concentration showed that the basicity affects the phosphate removal, and when the basicity is high, the phosphate removal is reduced, potentially due to the effect of the aluminum bond vibration. Sample R with the highest amount of aluminium and lowest basicity showed better phosphate removal ability than the other two samples with lower aluminium. The slag samples, however, added varying concentrations of toxic metals Al, Cd, Co and Hg into the treated water, which will need to be further conditioned by dilution with unpolluted water or other treatments before disposal or re-use.
